# Building factorial prompt matrices using query targets and configurable factor classes: A methods framework for conversational AI response behavior research

**DOI:** 10.1016/j.mex.2026.104062

**Published:** 2026-07-24

**Authors:** Kevin Corti, Meghan Leaver

**Affiliations:** Jebel Corp., New York, NY, United States

## Abstract

The term “factorial prompt matrix” (FPM) names and formalizes the emerging yet unstandardized practice of creating data structures that organize systematically varied prompts used to elicit responses from conversational AI (CAI) products. We introduce a methods framework for designing and implementing FPMs that, though broadly applicable, is primarily intended for social and behavioral scientists interested in controlled observational or experimental research on CAI response behavior.

FPMs start from a principled sampling spine of CAI query “target” items that are then crossed with configurable factors drawn from four classes: (1) the large language model (LLM), (2) the product-system, (3) the user context, and (4) the query design. This produces a factorial grid of distinct prompts that can be passed to CAIs (e.g., using APIs). Using the ICD-11 (International Classification of Diseases, Eleventh Revision) as a spine for generating medical prompts, we demonstrate use of FPMs to analyze CAI information retrieval behaviors such as web search invocation.

Key methodological contributions:

A formalized data structure (FPM) for systematically constructing and deploying prompts to CAIs.

A modular, four-class schema for conceptually organizing the configurable factors of a CAI prompting protocol.

Demonstration of FPMs across single-factor and multi-factor observational and experimental research designs.

## Graphical abstract

**Figure:**
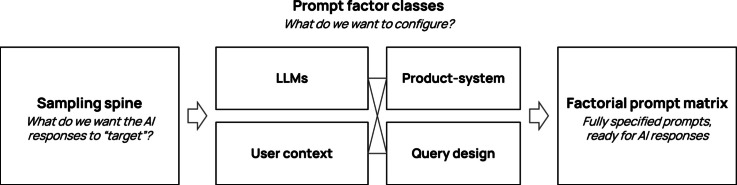

## Specifications table

**Table:** 

**Subject area**	Computer science
**More specific subject area**	Large language models
**Name of your method**	Factorial prompt matrices
**Name and reference of original method**	Not applicable
**Resource availability**	github.com/jebeldata/fpm-demo

## Background

Conversational AI (CAI) products built from large language models (LLMs) can give consequentially different responses to similar prompts based on mechanisms ranging from the obvious to the subtle to the unknowable [[1], [2], [3]].

Recent years have seen a marked rise in research comparing response variance between and within specific CAIs [1,4,5]. Much of this research shares a common design feature whereby elements of a CAI prompting protocol are systematically altered to observe whether doing so elicits meaningful response behavior change.

We make three pertinent observations about this empirical landscape.

First, CAI response behavior research designs are often either *one-factor-at-a-time* (OFAT) designs, which test a single factor at different factor *levels*, e.g., [6] or *factorial* designs, which test combinations of multiple factors and their levels. e.g., [3].

Second, while CAI APIs and user interfaces offer different means to toggle settings (e.g., turning CAI tools on) and invoke context or directives (e.g., via system instructions), such mechanics are not necessarily an expression of how factors should be conceptually classified.

Third, many of the techniques used to generate prompts according to factor specifications appear to be methods of programmatic convenience (which is not a criticism). However, the CAI field as a whole, and in particular researchers who are just entering it, may benefit from straightforward protocols for designing implementation-ready data structures that organize factor relationships and prompts. (Note: our use of the term “prompt” refers to the entire factorial configuration used to elicit a response from a CAI – not just a particular query or instruction).

This paper offers a methods framework that attempts to reconcile these observations. It is intended to be modular and flexible across CAI research domains. The demonstrations we provide reflect our particular interest in how CAIs answer medical prompts.

Our framework suggests that factors amenable to configuration by CAI researchers fall into one of four classes:

1. The *LLM* class, containing individual LLMs that can be accessed for analysis. e.g., [6,7].
2. The *product-system* class, containing the parameters that shape LLM behavior within a particular deployment context independent of the user context and individual queries. e.g., [8].
3. The *user context* class, containing the parameters that inform the product-system and LLM as to who the user is, what their goals are, their interaction history, and so on, independent of individual queries. e.g., [9].
4. The *query design* class, containing all possible configurable characteristics of a query that combine to encode an intention for the CAI to interpret during a single turn in interaction. e.g., [3].

Factors from these classes can be crossed with elements from a principled sampling spine of query targets, producing a *factorial prompt matrix* (FPM). FPMs are formalizations of the data structures described above and contain all combinations of factor levels and query targets desired by the researcher. FPMs can be used in OFAT and factorial CAI response behavior research, whether observational or experimental, exploratory or hypothesis-driven.

## Method details

### The basics of a factorial prompt matrix

The FPMs we employ are flattened dataframes (i.e., structures where rows contain unique prompt factor combinations) which can be stored in spreadsheets, .csv files, or queryable databases, and operationalized using analytics software or statistical computing environments like *R* or Python.

The construction logic underlying an FPM is straightforward. First, as just alluded to, the researcher identifies a list of query targets to serve as a sampling spine. This can be thought of as the core set of objects that the researcher seeks to observe CAI responses to. Next, the researcher defines factors drawn from one or more of the four classes mentioned above (and described in more detail below) relevant to their research goals. These factors are then crossed to produce factorial combinations of interest, which can range from a fully Cartesian crossing (i.e., all possible combinations of all factor levels) to more constrained or hypothesis-specific combinations. Additional metadata can be added as columns where relevant to give more context to each record. The result is a pre-response-elicitation FPM containing the fully designed query text in one column and the factor level values for the prompt in adjacent columns.

Pre-response-elicitation FPMs can be passed to CAIs (e.g., via APIs) and then subsequently used in data post-processing and formal analysis. Pre-response-elicitation FPMs can also serve as templates — placeholder structures for future research or from which derivative FPMs can be formed.

### The sampling spine

As mentioned above, sampling spines are the foundational component of an FPM. These can, for example, contain a list of keywords, a corpus of naturally observed user queries, a battery of questions or phrases, or items drawn from a pre-defined taxonomy.

Every row in an FPM ties back to an individual element within the spine. The spine is therefore a consequential early design decision and affects the degree of generalizability and ecological validity of the data ultimately gathered from the FPM. Ad hoc spines may suffice for narrow demonstrations and early methodological stress-testing, but formal research endeavors should contain spines that are meaningful representations of a population of CAI query targets relevant to the phenomena under investigation.

The ICD-11 (International Classification of Diseases, Eleventh Revision) [10] is an especially strong sampling spine for our use case given our interest in studying how CAIs respond to health and biomedical prompts. It is globally applied, supports multiple languages, and is regularly maintained and updated as medical knowledge evolves. Its hierarchical structure is conducive to granular sampling (e.g., drawing conditions from a specific disease category) and broad sampling (e.g., drawing conditions from multiple disease categories or the entire taxonomy). See Table 1 for a basic illustrative sampling spine composed of the two main forms of diabetes mellitus classified in the ICD-11.

**Table 1:** A basic two-item sampling spine drawn from the ICD-11.

Spine category	Item title
Diabetes mellitus	Type 1 diabetes mellitus
Diabetes mellitus	Type 2 diabetes mellitus

*Note*. The Item title column contains the FPM spine "target" while the remaining columns are metadata providing context.

### FPM factor classes

In our early experimentation and methodological development, we have identified four distinct classes of factors that can be operationalized in FPMs for CAI behavior research. Future work may refine this classification schema to identify a more granular or hierarchical classification structure. For now, however, and for the purposes of this exposition of FPMs, the current approach strikes the appropriate balance between exhaustiveness and mutual exclusivity.

**The LLM class.** The LLM factor class identifies language models that can be invoked to generate CAI responses. Individual LLMs selected by the researcher for a given FPM are instances of this class. An FPM’s spine can be crossed with as many individual LLMs as the researcher has hypotheses about, interest in, and access to (See Table 2 for an example LLM factor crossed with Table 1).

**Table 2:** The sampling spine from Table 1 Crossed with a two-level LLM factor designating LLMs.

Item title	LLM	LLM family
Type 1 diabetes mellitus	gpt-5.4-nano	OpenAI - GPT
Type 1 diabetes mellitus	gpt-5.4-mini	OpenAI - GPT
Type 2 diabetes mellitus	gpt-5.4-nano	OpenAI - GPT
Type 2 diabetes mellitus	gpt-5.4-mini	OpenAI - GPT

*Note*. The LLM column contains the factor level, while spine items represent sampling units.

**The product-system class.** In common parlance, LLMs and CAI products are often spoken of interchangeably. But there is a critical distinction between them that is highly relevant to CAI behavior research. Base LLMs are almost never directly experienced by users outside of specialized research or development contexts. Instead, CAIs (and other products built from LLMs) can best be thought of as product-systems wrapped around a particular LLM (or LLMs), with the user’s experience occurring at the product-system level (see [11] for further context on the nested scales at which users experience product-systems).

CAI product-systems contain a range of factors governing how a CAI behaves in a production environment, including brand policy, legal and regulatory guardrailing, security features, user experience design system implementations, compute optimization logic, as well as instructions for how the system should interpret and interact with other infrastructure or applications to which it is connected. Most companies that create CAIs never expose or afford the user control over sensitive or confidential parameters of the product-system. That being said, the apps, web interfaces, and APIs through which users interact with CAIs often afford some configuration control over this layer.

Examples of configurable product-system factors include things like system-level instructions (i.e., that govern how the CAI should behave across all queries, or how the CAI should interpret the application context in which it is deployed), tool toggles (i.e., the applications or features that the CAI can make use of as part of its interaction with the user), token constraints (i.e., compute or cost thresholds), and temperature settings (i.e., the degree of randomness in CAI response generation).

A further distinction we make here is conceptual rather than technical: certain configurable product-system factors are often toggled through the same API functions and user interface mechanisms as user context factors (discussed in the following subsection). That being said, we draw a line between the two in that we consider product-system factors to be those that shape how CAIs behave independent of who any particular user or account is.

Determining product-system configuration factors for multi-LLM FPMs can be complex. This is because while some standardization exists across LLMs and developers on product-system terminology and configuration mechanisms, there is also divergence. As one example, OpenAI and Anthropic currently use different parameter structures for *reasoning effort*, which is a configurable setting that governs the depth of internal reasoning performed by the CAI [12,13]. FPM designs comparing multiple LLMs should therefore take into account these differences and schematize accordingly. Reconciling contrasting product-system factors across different LLMs and CAI products falls outside the scope of this article. (See Table 3 for an example product-system factor crossed with Table 2).

**Table 3:** Table 2 Crossed with a two-level product-system factor designating system instructions.

Item title	LLM	Product-system: system instruction	System instruction label
Type 1 diabetes mellitus	gpt-5.4-nano	“You are operating within a specialized biomedical literature search engine.”	Biomedical search engine
Type 1 diabetes mellitus	gpt-5.4-nano	“You are operating within a consumer smartphone chatbot application”	Smartphone chatbot app
Type 1 diabetes mellitus	gpt-5.4-mini	“You are operating within a specialized biomedical literature search engine.”	Biomedical search engine
Type 1 diabetes mellitus	gpt-5.4-mini	“You are operating within a consumer smartphone chatbot application”	Smartphone chatbot app
Type 2 diabetes mellitus	gpt-5.4-nano	“You are operating within a specialized biomedical literature search engine.”	Biomedical search engine
Type 2 diabetes mellitus	gpt-5.4-nano	“You are operating within a consumer smartphone chatbot application”	Smartphone chatbot app
Type 2 diabetes mellitus	gpt-5.4-mini	“You are operating within a specialized biomedical literature search engine.”	Biomedical search engine
Type 2 diabetes mellitus	gpt-5.4-mini	“You are operating within a consumer smartphone chatbot application”	Smartphone chatbot app

*Note.* We now have a 2 × 2 factorial research design. Metadata columns are suppressed to reduce redundancy; in practice, all columns would be retained.

**The user context class.** To restate, we draw a conceptual line between product-system configurations and user context factors, with the latter being those which personalize behavior toward various known attributes of the user.

User context factors open rich possibilities for social psychological research. Examples of user context factors include user personas (i.e., a characterization of the user's background, demographics, expertise, or role), situational attributes (e.g., user journeys or goals, reference documents, and other use case information), and historical context (e.g., user interaction history and retained memories). In CAI behavior research, the content of these factors is typically generated by the researcher or borrowed from established methods to simulate real user contexts, though FPMs can also accommodate actual user personal data in situations where users have consented to their data being used by the researcher.

Given the degree of user personalization built into many CAI products, user context configurations are an important lever in determining the ecological validity of the research being conducted. We should expect certain qualities of CAI responses to medical questions, for instance, to vary meaningfully depending on whether they are posed by a user known to be a physician versus one known to have no association with the medical profession. Exploration of this variance is exactly the type of research FPMs are well-suited for. (See Table 4 for an example user context factor crossed with Table 3).

**Table 4:** Table 3 Crossed with a two-level user context factor designating user personas.

Item title	LLM	Product-system: system instruction label	User context: persona
Type 1 diabetes mellitus	gpt-5.4-nano	Biomedical search engine	“User is a 24-year-old medical student”
Type 1 diabetes mellitus	gpt-5.4-nano	Biomedical search engine	“User is a 55-year-old stand-up comic”
Type 1 diabetes mellitus	gpt-5.4-nano	Smartphone chatbot app	“User is a 24-year-old medical student”
Type 1 diabetes mellitus	gpt-5.4-nano	Smartphone chatbot app	“User is a 55-year-old stand-up comic”
Type 1 diabetes mellitus	gpt-5.4-mini	Biomedical search engine	“User is a 24-year-old medical student”
Type 1 diabetes mellitus	gpt-5.4-mini	Biomedical search engine	“User is a 55-year-old stand-up comic”
Type 1 diabetes mellitus	gpt-5.4-mini	Smartphone chatbot app	“User is a 24-year-old medical student”
Type 1 diabetes mellitus	gpt-5.4-mini	Smartphone chatbot app	“User is a 55-year-old stand-up comic”
Type 2 diabetes mellitus	gpt-5.4-nano	Biomedical search engine	“User is a 24-year-old medical student”
Type 2 diabetes mellitus	gpt-5.4-nano	Biomedical search engine	“User is a 55-year-old stand-up comic”
Type 2 diabetes mellitus	gpt-5.4-nano	Smartphone chatbot app	“User is a 24-year-old medical student”
Type 2 diabetes mellitus	gpt-5.4-nano	Smartphone chatbot app	“User is a 55-year-old stand-up comic”
Type 2 diabetes mellitus	gpt-5.4-mini	Biomedical search engine	“User is a 24-year-old medical student”
Type 2 diabetes mellitus	gpt-5.4-mini	Biomedical search engine	“User is a 55-year-old stand-up comic”
Type 2 diabetes mellitus	gpt-5.4-mini	Smartphone chatbot app	“User is a 24-year-old medical student”
Type 2 diabetes mellitus	gpt-5.4-mini	Smartphone chatbot app	“User is a 55-year-old stand-up comic”

*Note.* We now have a 2 × 2 × 2 factorial research design. Metadata columns are suppressed to reduce redundancy; in practice, all columns would be retained.

**The query design class.** The query design class encompasses all possible configurations of how content and intention are expressed by a user via a query during a single turn of interaction with a CAI.

Within our FPM framework, a column containing the queries to be passed to a CAI is generated by taking the spine item for each record and transforming it into a final form based on chosen design factors. For instance, the researcher may want to use a single, unchanging query template across spine items (e.g., “What are treatments for Type 1 diabetes mellitus?”, “What are treatments for Type 2 diabetes mellitus?”, and so on). Or they may want to systematically manipulate elements of the query design based on a hypothesis or exploratory interest (e.g., by invoking a multi-level topic factor: “What are *treatments for* Type 1 diabetes mellitus?” versus “What are *symptoms of* Type 1 diabetes mellitus?”).

The query design factors that we have explored in our early research include *topic* manipulations like that just described which orient the CAI toward developing a response to a particular aspect of the spine item. We have explored explicit *directives* – language that instructs the CAI as to how its response should be framed or what knowledge sources to draw from; these have been instrumented both as query prefixes (e.g., “*According to the CDC*, what are risk factors for Type 2 diabetes mellitus?”) as well as query suffixes (e.g., “What are risk factors for Type 2 diabetes mellitus, *based on the latest research and clinical guidelines*?”). We have also investigated *parlance transposition*, whereby we transform a spine item from one parlance to a semantically equivalent alternative (e.g., clinical/ICD terminology: “What are symptoms of *Streptococcal pharyngitis*?” versus layperson terminology: “What are symptoms of *strep throat*?”). Additionally, we have manipulated full query language (e.g., English versus Spanish). These examples are by no means exhaustive given the considerable degrees of freedom available within this factor class.

Once final decisions about query design characteristics have been made, generating the final query for each record in an FPM can be performed programmatically. We have used an approach whereby we create the individual query design variations we desire through use of wildcard substrings in a query phrase template, into which spine items and the other query elements we wish to manipulate can be inserted using string functions (e.g., “What is %X%?” gets programmatically transformed to: “What is *Type 1 diabetes mellitus*?”, “What is *Streptococcal pharyngitis*?”, and so on for all spine items).

As a final point, query design characteristics are arguably the most direct lever for ecological validity in FPM design. Real-world CAI users vary enormously in how they express themselves in terminology, language, inclination to use explicit directives, and so on. Systematic manipulation of these characteristics within our framework allows a researcher to approximate that diversity in a controlled way. (See Table 5 for an example FPM consisting of two crossed query design factors).

**Table 5:** An example 2 × 2 factorial prompt matrix consisting of two crossed query design factors.

Item title	Query design: topic category	Query design: directive	Example query
Type 1 diabetes mellitus	Risk factors	*None supplied*	“What are risk factors for Type 1 diabetes mellitus?”
Type 1 diabetes mellitus	Risk factors	Clinical guidelines	“According to clinical guidelines, what are risk factors for Type 1 diabetes mellitus?”
Type 1 diabetes mellitus	Treatments	*None supplied*	“What are treatments for Type 1 diabetes mellitus?”
Type 1 diabetes mellitus	Treatments	Clinical guidelines	“According to clinical guidelines, what are treatments for Type 1 diabetes mellitus?”
Type 2 diabetes mellitus	Risk factors	*None supplied*	“What are risk factors for Type 2 diabetes mellitus?”
Type 2 diabetes mellitus	Risk factors	Clinical guidelines	“According to clinical guidelines, what are risk factors for Type 2 diabetes mellitus?”
Type 2 diabetes mellitus	Treatments	*None supplied*	“What are treatments for Type 2 diabetes mellitus?”
Type 2 diabetes mellitus	Treatments	Clinical guidelines	“According to clinical guidelines, what are treatments for Type 2 diabetes mellitus?”

## Method validation

What follows are five demonstrations of the design and use of FPMs for observing CAI response behavior variation in relation to different configurations of factors from the classes outlined above.

### A note on the LLMs used in our demonstrations

All of the demonstrations in this paper use OpenAI GPT family LLMs accessed via OpenAI’s Responses API [14]. As the purpose of this paper is to showcase our FPM and factor class framework rather than make strong claims about particular models, using LLMs from a single developer is a deliberate choice that keeps code concise and avoids having to manage multiple API integrations.

The three specific GPT LLMs used across our demonstrations are gpt-5-nano, gpt-5.4-nano, and gpt-5.4-mini, all of which were designated by OpenAI as being frontier models in May 2026 [15]. We chose not to include current frontier “high-end” GPT LLMs (e.g., gpt-5.5 or gpt-5.5-pro) in our demonstrations because such LLMs have significantly higher token costs and response latencies. In our determination, this would unnecessarily burden researchers interested in replicating our demonstrations without adding anything relevant to the goals of this paper given that, as will be shown, significant behavior variance is observable across the more economically efficient LLMs.

An important qualifier for interpreting our demonstrations is that in all cases where a product-system factor or user context factor is not explicitly configured, the CAI will behave according to OpenAI’s default parameters for that factor. This is the general behavior of CAIs. Some default settings are published by developers in publicly available documentation, while others — particularly those concerning legal guardrails, brand policy, and proprietary technology — are typically not publicly disclosed.

### A note on the general approach of our demonstrations

The ICD-11 contains thousands of individual medical conditions across more than twenty chapters of disease classes. Given that our goal with this paper is to present our methodological framework rather than make substantive claims about CAI medical competencies, our demonstrations use a restricted taxonomic sampling spine consisting of 60 individual *Mycoses* grouped within the ICD-11′s Chapter 1 on *Certain infectious or parasitic diseases*. In our judgement, 60 individual disease targets function as a sufficient sample for demonstration purposes. Additionally, we considered it methodologically preferable to develop a demonstration spine from all diseases within one particular subclass of the ICD-11 rather than draw a similar number of individual disease conditions from across multiple ICD-11 chapters. Also, readers need not have any expertise in mycoses or medical conditions more broadly to follow the implications of FPM methodology.

The dependent measures we selected for these demonstrations primarily concern observable information retrieval behaviors of the CAI as reflected in response outputs. In all demonstrations, the CAI had access to a web search tool — enabled via API configuration — but was not required to use it; invoking it was left to the CAI's discretion [16]. We are interested in web search invocation as a grounding behavior for medical queries because it reveals the rate at which a CAI draws on external sources for supporting information, and how that rate varies across factor combinations. We therefore use web search invocation rate as a dependent variable across all demonstrations. In the fifth demonstration we add a second dependent measure: URL domain localization.

The supplementary files for this paper include a copy of the Python code executed using Google Colab to produce the FPM dataframes for each demonstration as well as elicit responses via OpenAI’s Responses API. Also included is the *R* code used for post-processing of the elicited data and statistical analysis.

### Demonstration 1: CAI web search invocation across three LLMs

Our first demonstration showcases a very basic FPM with only the model factor class being varied. It observes whether the rate of web search invocation changes significantly across three different LLMs: gpt-5-nano, gpt-5.4-nano, and gpt-5.4-mini. This is an OFAT experimental design, as one factor (LLM) is being compared across its different levels, with no other factors invoked.

Query construction for this demonstration uses a single phrase template (“What is %X%?”) crossed with each of the 60 individual mycoses conditions from the ICD-11. This produces 60 individual queries per LLM, resulting in 180 total FPM rows. (See Table 6).

**Table 6:** The structure of the FPM used in demonstration 1.

LLM	Example query
gpt-5-nano	“What is Basidiobolomycosis?”
gpt-5.4-nano	“What is Basidiobolomycosis?”
gpt-5.4-mini	“What is Basidiobolomycosis?”

*Note.* This table intentionally suppresses various FPM columns and rows beyond those corresponding to a single spine item for demonstration purposes. See supplementary files for code producing the full FPM used in this demonstration.

After collecting response data, we observed significant differences in web search invocation rates across models. Gpt-5-nano invoked web search in 75.0% of queries, gpt-5.4-mini in 20.0%, and gpt-5.4-nano in 0.0%. Pairwise comparisons with Bonferroni correction confirmed that each pairwise difference was statistically significant (all *p *< 0.01). (See Table 7).

**Table 7:** Statistical summary for demonstration 1.

LLM	N queries	Web search rate [95% CI]
gpt-5-nano	60	75.0% [62.8%, 84.2%]
gpt-5.4-nano	60	0.0% [0.0%, 6.0%]
gpt-5.4-mini	60	20.0% [11.8%, 31.8%]

*Note.* Confidence intervals computed using Wilson method. See supplementary files for the full statistical procedures used for this demonstration.

### Demonstration 2: CAI web search invocation as a function of supplied directive location

Our second demonstration explores whether web search invocation changes as a result of where within a prompt a specific query directive is placed. The directive used is “Consider using web search to ground your answer”. In condition 1, this directive is placed directly within the user query as a suffix. In condition 2, the directive is used as a Responses API developer instruction (a product-system configuration). In condition 3, the directive is used as a Responses API system instruction (a product-system configuration). Condition 4, meanwhile, is a baseline condition in which the directive is not supplied anywhere. One LLM is held constant across all four conditions (gpt-5.4-nano), resulting in a partial factorial design.

As with the first demonstration, query construction for this demonstration uses a single phrase template (“What is %X%?”) crossed with each of the 60 individual mycoses conditions from the ICD-11, producing 60 individual queries per condition. (See Table 8).

**Table 8:** The structure of the FPM Used in demonstration 2.

Condition label	LLM	Query design: directive suffix	Product-system: developer instruction	Product-system: system instruction	Example query
1	gpt-5.4-nano	“Consider using web search to ground your answer.”	*None supplied*	*None supplied*	“What is Basidiobolomycosis? Consider using web search to ground your answer.”
2	gpt-5.4-nano	*None supplied*	“Consider using web search to ground your answer.”	*None supplied*	“What is Basidiobolomycosis?”
3	gpt-5.4-nano	*None supplied*	*None supplied*	“Consider using web search to ground your answer.”	“What is Basidiobolomycosis?”
4	gpt-5.4-nano	*None supplied*	*None supplied*	*None supplied*	“What is Basidiobolomycosis?”

*Note.* This table intentionally suppresses various FPM columns and rows beyond those corresponding to a single spine item for demonstration purposes. See supplementary files for code producing the full FPM used in this demonstration.

Our results showed that web search invocation varied considerably based on the location of the directive. When the directive to consider using web search was entirely absent (condition 4), gpt-5.4-nano never (0.0%) invoked web search – thus replicating what was observed of the same LLM in our first demonstration. Condition 1, meanwhile, which placed the directive within the user query, showed maximum (100.0%) web search invocation. Condition 2, in which the directive was supplied as a developer instruction, showed 36.7% web search invocation. Condition 3, in which the directive was supplied as a system instruction, showed 41.7% web search invocation. All pairwise comparisons (with Bonferroni correction) were statistically significant (all *p *< 0.001) except for the developer instruction versus system instruction contrast. (See Table 9).

**Table 9:** Statistical summary for demonstration 2.

Condition label	LLM	Directive location	N queries	Web search rate [95% CI]
1	gpt-5.4-nano	Query design: User query suffix	60	100.0% [94.0%, 100.0%]
2	gpt-5.4-nano	Product-system: Developer instruction	60	36.7% [25.6%, 49.3%]
3	gpt-5.4-nano	Product-system: System instruction	60	41.7% [30.1%, 54.3%]
4	gpt-5.4-nano	Baseline (Directive absent)	60	0.0% [0.0%, 6.0%]

*Note.* Confidence intervals computed using Wilson method. See supplementary files for the full statistical procedures used for this demonstration.

### Demonstration 3: CAI web search invocation as a function of the LLM and reasoning effort

Our third demonstration is our first full factorial design, crossing two LLMs (gpt-5.4-nano and gpt-5.4-mini) with two levels of reasoning effort: "low" and "none". As alluded to above, reasoning effort is a product-system configuration that governs the volume of computational resources that the CAI allocates to internal reasoning processes before generating a response. In practical terms, reasoning effort set to “none” means the LLM will generate responses directly without internal deliberation; when set to “low”, the LLM will use some computational resources to optimize a response, but not an extensive amount.

As with the first two demonstrations, query construction for this demonstration uses a single phrase template (“What is %X%?”) crossed with each of the 60 individual mycoses conditions from the ICD-11, producing 60 individual queries per condition. (See Table 10).

**Table 10:** The structure of the FPM used in demonstration 3.

LLM	Product-system: reasoning level	Example query
gpt-5.4-nano	None	“What is Basidiobolomycosis?”
gpt-5.4-nano	Low	“What is Basidiobolomycosis?”
gpt-5.4-mini	None	“What is Basidiobolomycosis?”
gpt-5.4-mini	Low	“What is Basidiobolomycosis?”

*Note.* This table intentionally suppresses various FPM columns and rows beyond those corresponding to a single spine item for demonstration purposes. See supplementary files for code producing the full FPM used in this demonstration.

Results indicated that web search invocation varied considerably based on model and reasoning effort level. Gpt-5.4-nano showed no web search invocation at either reasoning level, whereas gpt-5.4-mini invoked web search for 18.3% of queries when reasoning was set to “none” relative to 93.3% of queries when reasoning was “low”. All pairwise comparisons (with Bonferroni corrections) were statistically significant (all *p *< 0.01) except for the contrast between reasoning levels for gpt-5.4-nano. (See Table 11).

**Table 11:** Statistical summary for demonstration 3.

LLM	Product-system: reasoning level	N queries	Web search rate [95% CI]
gpt-5.4-nano	None	60	0.0% [0.0%, 6.0%]
gpt-5.4-nano	Low	60	0.0% [0.0%, 6.0%]
gpt-5.4-mini	None	60	18.3% [10.6%, 29.9%]
gpt-5.4-mini	Low	60	93.3% [84.1%, 97.4%]

*Note.* Confidence intervals computed using Wilson method. See supplementary files for the full statistical procedures used for this demonstration.

### Demonstration 4: CAI web search invocation as a function of reasoning effort, user persona, and query topic

For our fourth demonstration we invoke three factors simultaneously. First, we configure the reasoning effort level as with the previous demonstration to be either “none” or “low”. Next, we introduce a two-level user persona factor established via the Responses API developer instruction mechanism: “Known user persona: user often conducts literature reviews for scientific papers” (labeled as "Biomedical researcher" in our FPM) versus no persona defined. Finally, we explore a two-level query topic factor (general description: “What is %X%?” versus risk factors: “What are risk factors for %X%?”).

For this demonstration, the LLM is fixed as gpt-5.4-nano across all conditions, producing a 2 × 2 × 2 full factorial design targeting each of the 60 individual mycoses conditions from the ICD-11. (See Table 12).

**Table 12:** The structure of the FPM used in demonstration 4.

LLM	Product-system: reasoning level	User context: persona	Query design: topic	Example query
gpt-5.4-nano	None	Biomedical researcher	General description	“What is Basidiobolomycosis?”
gpt-5.4-nano	None	Biomedical researcher	Risk factors	“What are risk factors for Basidiobolomycosis?”
gpt-5.4-nano	None	*None supplied*	General description	“What is Basidiobolomycosis?”
gpt-5.4-nano	None	*None supplied*	Risk factors	“What are risk factors for Basidiobolomycosis?”
gpt-5.4-nano	Low	Biomedical researcher	General description	“What is Basidiobolomycosis?”
gpt-5.4-nano	Low	Biomedical researcher	Risk factors	“What are risk factors for Basidiobolomycosis?”
gpt-5.4-nano	Low	*None supplied*	General description	“What is Basidiobolomycosis?”
gpt-5.4-nano	Low	*None supplied*	Risk factors	“What are risk factors for Basidiobolomycosis?”

*Note.* This table intentionally suppresses various FPM columns and rows beyond those corresponding to a single spine item for demonstration purposes. See supplementary files for code producing the full FPM used in this demonstration.

Web search invocation rates were again observed to be highly dependent on factor combinations. All conditions with reasoning set to “none” showed 0.0% web search invocation regardless of query topic and persona. Within the conditions where reasoning was set to “low”, query topic was the primary differentiator as general description queries produced just a 1.7% web search invocation rate (for both persona conditions) versus 70.0% for risk factor queries with no established persona and a directional (albeit statistically insignificant) increase to 76.7% for risk factor queries when the persona was applied. (See Table 13).

**Table 13:** Statistical summary for demonstration 4.

LLM	Product-system: reasoning level	User context: persona	Query design: topic	N queries	Web search rate [95% CI]
gpt-5.4-nano	None	Biomedical researcher	General description	60	0.0% [0.0%, 6.0%]
gpt-5.4-nano	None	Biomedical researcher	Risk factors	60	0.0% [0.0%, 6.0%]
gpt-5.4-nano	None	*None supplied*	General description	60	0.0% [0.0%, 6.0%]
gpt-5.4-nano	None	*None supplied*	Risk factors	60	0.0% [0.0%, 6.0%]
gpt-5.4-nano	Low	Biomedical researcher	General description	60	1.7% [0.3%, 8.9%]
gpt-5.4-nano	Low	Biomedical researcher	Risk factors	60	76.7% [64.6%, 85.6%]
gpt-5.4-nano	Low	*None supplied*	General description	60	1.7% [0.3%, 8.9%]
gpt-5.4-nano	Low	*None supplied*	Risk factors	60	70.0% [57.5%, 80.1%]

*Note*. Confidence intervals computed using Wilson method. See supplementary files for the full statistical procedures used for this demonstration.

### Demonstration 5: CAI web search invocation and source localization as a function of query language, with repeated measures

Our final demonstration uses a basic OFAT design, examining whether web search invocation rates vary across three query languages (English versus Spanish versus Kazakh).

Query design for this demonstration uses a single phrase template (“What is %X%?”) translated into each of the three languages using Google Translate and crossed with each of the mycoses spine items using ICD-11 translations. This demonstration employs a repeated-measures design, as each spine item-language prompt combination is repeated three times. This produces 540 total FPM rows (60 mycoses conditions x 3 languages x 3 repetitions), all submitted to gpt-5-nano. (See Table 14).

**Table 14:** The structure of the FPM used in demonstration 5.

LLM	Query design: language	Repetitions	Example query
gpt-5-nano	English	3	“What is Basidiobolomycosis?”
gpt-5-nano	Spanish	3	“¿Qué es la Basidiobolomicosis?”
gpt-5-nano	Kazakh	3	“Базидиоломикоз дегеніміз не?”

*Note.* This table intentionally suppresses various FPM columns and rows beyond those corresponding to a single spine item for demonstration purposes. See supplementary files for code producing the full FPM used in this demonstration.

In addition to web search invocation, this demonstration examines URL source localization as a second dependent variable — specifically, whether the language of a query shapes the geographic or linguistic origin of sources the CAI retrieves. We operationalized localization at the web domain level using country-code top-level domains as a heuristic. For example, .es and .mx were among the indicators we used for Spanish-localized sources, while .kz was among the indicators of Kazakh-localized sources. Full details of the domain classification procedure are provided in the supplementary *R* code.

Results showed that web search invocation rates varied considerably across query languages. Spanish queries showed the highest overall rate (80.0%), followed by English (72.2%), and Kazakh (55.6%). Pairwise comparisons (with Bonferroni corrections) following a mixed-effects logistic regression model accounting for repeated measures confirmed that Kazakh queries produced significantly lower web search invocation rates compared to both English (*p *< 0.01) and Spanish (*p *< 0.001), while English and Spanish queries did not significantly differ from each other. (See Table 15).

**Table 15:** Statistical summary for demonstration 5.

LLM	Query design: language	N queries	Web search rate [95% CI]	Unique URLs retrieved	Localized URLs
gpt-5-nano	English	180	72.2% [65.3%, 78.2%]	335	*NA (reference)*
gpt-5-nano	Spanish	180	80.0% [73.6%, 85.2%]	447	7
gpt-5-nano	Kazakh	180	55.6% [48.3%, 62.6%]	259	0

*Note.* Confidence intervals computed using Wilson method. See supplementary files for the full statistical procedures used for this demonstration. URL domain localization was assessed for Spanish and Kazakh language domains specifically. English-language domains were treated as the reference category and are not counted as localized.

Of the 790 globally unique URLs retrieved across all conditions, we classified 7 URLs as having Spanish-localized domains, and 0 for Kazakh.

## In summary

Taken together, our five demonstrations, all using OpenAI GPT family LLMs, demonstrate that FPMs can be used for controlled manipulation and analysis of CAI behavior variance via simple and multi-factorial research designs as well as repeated-measures implementation. Though our research interests and these demonstrations concern CAI response behavior toward medical queries, the FPM framework can extend across research domains, and we encourage researchers to explore and validate the framework in their own fields. We plan to continue development of the FPM framework, including scaling it to involve longitudinal research, as well as further iterating upon the four factor-class structure.

## Limitations

We acknowledge that findings from the LLMs in our demonstrations may not generalize to all OpenAI models or comparable LLMs from other providers. Researchers should refer to relevant API resources and other published documentation to understand how implementation mechanisms and configuration affordances may differ across LLMs.

As our demonstration spine was restricted to 60 individual mycoses disease conditions in the ICD-11 for demonstration purposes, this design decision may have resulted in data that only generalizes to a limited degree beyond the particular subclass of the ICD from which the spine was extracted. Researchers interested in making broader claims about CAI responses to medical queries are encouraged to develop sampling spines from wider portions of the ICD and other medical taxonomies.

Combinatorial explosion is always a risk when designing factorial experiments or when using large sampling spines. As such, very large FPMs are likely to incur higher API costs, longer API processing times, and may require more sophisticated cost control and parallelization strategies.

Lastly, CAI product development is a rapidly evolving technological space. AI providers release new models and updates to existing models at a pace that far eclipses traditional research publication cycles. Such velocity, combined with the continually evolving nature of product-system governance acting "under the hood" of CAI products, makes CAI behavior an ever-moving target. Our hope is that the FPM framework nonetheless makes CAI research more tractable, standardized, and approachable for AI specialists and behavioral scientists broadly.

## Ethics statements

The authors declare that the research and preparation of this manuscript complied with the ethical guidelines of the journal. Specifically, the work did not involve human subjects or animal experiments and did not involve collection of data from social media platforms.

## Related research article

None.

## Declaration of generative AI use

The authors disclose that portions of the Python code used for data-elicitation were produced with the assistance of Google’s Gemini code assistant features in Google Colab, especially for debugging, consistency, formatting, and readability. OpenAI’s ChatGPT code assistant features were used for portions of Python code specific to the use of OpenAI’s Responses API. Portions of the *R* code used for data analysis were produced with the assistance of Anthropic's Claude, particularly for debugging, consistency, formatting, and readability. Claude was also utilized in the drafting of the main manuscript in a manner consistent with the editorial policies of the publisher for the purposes of improving readability, grammar, concision, and cohesion.

After using these tools, the authors reviewed and edited the content as needed and take full responsibility for the content of the published article.

## Supplementary material *and/or* additional information

Two supplementary files are available for this article (at github.com/jebeldata/fpm-demo): a Python (.py) file reflecting the data-elicitation workflow for the article’s five demonstrations, and an *R* (.R) file documenting the data analysis.

## CRediT authorship contribution statement

**Kevin Corti:** Conceptualization, Methodology, Software, Validation, Formal analysis, Investigation, Resources, Data curation, Writing – original draft, Visualization, Supervision. **Meghan Leaver:** Conceptualization, Methodology, Investigation, Resources, Writing – review & editing, Supervision.

## Declaration of competing interest

The authors declare the following financial interests/personal relationships which may be considered as potential competing interests:

Kevin Corti reports a relationship with Jebel Corp. that includes: board membership, employment, and equity or stocks. Meghan Leaver reports a relationship with Jebel Corp. that includes: board membership, employment, and equity or stocks.

## Data Availability

Replication code and analysis files can be accessed for free at github.com/jebeldata/fpm-demo.
